# Association between platelet to white blood cell ratio and post-stroke epilepsy in patients with acute ischemic stroke: a multicenter retrospective cohort study

**DOI:** 10.3389/fneur.2026.1870448

**Published:** 2026-07-29

**Authors:** Shuaibin Huang, Xuejuan Wu, Ke Xu

**Affiliations:** 1 Department of Neurosurgery, Shantou Central Hospital, Shantou, China; 2 Neurocritical Care Unit, Shantou Central Hospital, Shantou, China

**Keywords:** acute ischemic stroke, biomarker, nonlinear relationship, platelet to white blood cell ratio, post-stroke epilepsy

## Abstract

**Background:**

Post-stroke epilepsy (PSE) is a significant complication following acute ischemic stroke (AIS). The platelet to white blood cell (WBC) ratio (PWR) has emerged as a prognostic marker in stroke populations, but its association with PSE remains unknown. This study investigated the relationship between PWR and PSE incidence in AIS patients.

**Methods:**

This multicenter retrospective cohort study included 21,459 patients from four hospitals. PWR was calculated as platelet count divided by WBC count. The primary outcome was PSE incidence within 1 year post-stroke. Multivariable logistic regression, restricted cubic spline analysis, and subgroup analyses were performed. Receiver operating characteristic curves and DeLong’s test compared the predictive performance of PWR with its individual components.

**Results:**

Higher PWR was independently associated with lower PSE risk [odds ratio (OR) per 1-standard deviation increase: 0.214; 95% confidence interval (CI): 0.186–0.245; *p* < 0.001]. A dose–response relationship was observed across quartiles (quartile 4 vs. quartile 1: OR 0.033; 95% CI: 0.018–0.059; *p* for trend <0.001). Restricted cubic spline analysis revealed a significant nonlinear relationship between PWR and PSE risk (*p* for nonlinearity <0.001). A two-piecewise linear regression model further identified a statistically significant change in slope at an inflection point of 20.31. Subgroup analyses revealed significant interactions for diabetes, carotid plaque, paraventricular involvement, gender, and anterior circulation involvement. PWR demonstrated superior predictive performance (area under the curve: 0.879) compared to platelet or WBC alone.

**Conclusion:**

Higher PWR was independently associated with lower PSE risk in AIS patients, with a significant nonlinear relationship. PWR may serve as a simple, inexpensive biomarker for early PSE risk stratification.

## 1 Introduction

Stroke is a leading cause of death and long-term disability worldwide, with acute ischemic stroke (AIS) accounting for approximately 80% of all stroke cases (1, 2). Among the various complications following AIS, post-stroke epilepsy (PSE) represents a significant clinical challenge, affecting 5–20% of stroke survivors and contributing to increased morbidity, mortality, and reduced quality of life (3–5). The identification of reliable biomarkers for PSE risk stratification is essential for early intervention and improved patient outcomes.

The pathogenesis of PSE is complex and multifactorial, involving neuroinflammation, blood–brain barrier disruption, neuronal hyperexcitability, and aberrant synaptic reorganization following ischemic injury (6, 7). Systemic inflammatory responses play a crucial role in this process (8), with various inflammatory markers having been associated with post-stroke seizure risk (9, 10). Recently, composite inflammatory ratios derived from routine complete blood counts have gained attention as easily accessible prognostic markers in cerebrovascular diseases (9, 11, 12).

The platelet to white blood cell (WBC) ratio (PWR) is a novel composite marker that integrates information from both thrombopoietic and leukopoietic systems. Platelets and WBC are key players in inflammation, thrombosis, and tissue repair, and their balance may reflect the complex interplay between pro-inflammatory and anti-inflammatory pathways following ischemic injury. Previous studies have demonstrated the prognostic value of PWR in various AIS populations, showing associations with mechanical thrombectomy outcomes (13), stroke severity (14), and 90-day functional outcomes after intravenous thrombolysis (15). However, the relationship between PWR and PSE has not been previously investigated.

Regarding the individual components of PWR, both platelet and WBC have been independently associated with stroke outcomes and epilepsy risk in previous literature. Elevated WBC, as a marker of systemic inflammation, has been linked to poorer outcomes after stroke (16–18), and increased seizure susceptibility (19). Conversely, platelets contain and release various neurotrophic factors that may promote neuronal survival and tissue repair (20, 21), although their role in epileptogenesis remains incompletely understood. The PWR, by integrating these two opposing pathways, may provide a more comprehensive assessment of the inflammatory-thrombotic balance than either marker alone.

Therefore, this multicenter retrospective cohort study aimed to investigate the association between PWR and the incidence of PSE in patients with AIS.

## 2 Methods

### 2.1 Study design and population

This study was a secondary analysis of a previously published multicenter retrospective study (22). The original data were obtained from the Dryad database,^1^ which included patients with acute ischemic stroke (AIS) admitted to four hospitals in Chongqing, China, between June 2017 and July 2023 (23). The original study enrolled patients who were between 18 and 90 years of age and had a confirmed diagnosis of AIS requiring hospitalization. Patients were not eligible if they met any of the following criteria: a prior history of stroke or transient ischemic attack; the presence of conditions predisposing to epilepsy, such as traumatic brain injury, intracranial tumors, or cerebral vascular malformations; a previous diagnosis of epilepsy or use of antiseizure medications for any indication, including migraine or psychiatric disorders; or death occurring within 72 h of stroke onset (22). Patients without outpatient records who were unreachable by telephone, as well as those who died within 3 months of the stroke incident, were excluded during cohort construction. The final cohort comprised 21,459 patients. This study was conducted to investigate the association between the PWR and the incidence of PSE. The original study by Liu et al. (22) obtained ethical approval and informed consent prior to data collection and deposition. As this secondary analysis utilized fully anonymized, publicly accessible data, no additional IRB approval or informed consent was required, consistent with the policies of our institution and the Declaration of Helsinki.

#### 2.1.1 Standard stroke management protocol

For eligible patients presenting within the therapeutic time window, intravenous thrombolysis (alteplase) and/or endovascular thrombectomy were administered. For patients with non-cardioembolic AIS, standard practice included initiation of single antiplatelet therapy (aspirin 100–300 mg/day or clopidogrel 75 mg/day) within 24–48 h of onset. For patients with cardioembolic stroke (e.g., atrial fibrillation), anticoagulation (warfarin or direct oral anticoagulants) was typically initiated within 3–14 days post-stroke after exclusion of hemorrhagic transformation on follow-up imaging. In addition, all patients received guideline-directed statin therapy, blood pressure management, and glycemic control as part of standard secondary prevention. Importantly, while these center-level protocols were uniformly applied, individual-level data on specific drug selection, dosage, exact timing of initiation, and pre-admission medication use were not available in the original Dryad dataset and could therefore not be adjusted for in our analyses.

### 2.2 Variable definitions

#### 2.2.1 Exposure variable

PWR was calculated as the platelet count (×10^9^/L) divided by the WBC (×10^9^/L). For analysis, PWR was treated both as a continuous variable [per 1-standard deviation (SD) increase] and as a categorical variable (divided into quartiles, Q1–Q4).

#### 2.2.2 Outcome variable

The primary outcome was PSE incidence within 1 year following the index stroke. The diagnosis of PSE was established based on internationally recognized clinical standards (24). Specifically, PSE was diagnosed when patients met either of the following criteria: (1) two or more unprovoked (or reflex) seizures occurring >24 h apart, with the first seizure occurring >7 days after stroke onset (i.e., late seizure); or (2) a single unprovoked seizure occurring >7 days after stroke onset accompanied by clinical evidence—such as neuroimaging-confirmed structural lesion, epileptiform discharges on electroencephalography (EEG), or a clinical assessment by a neurologist—indicating a substantial risk of recurrence (24).

#### 2.2.3 Data collection and covariates

Clinical and laboratory data were extracted from hospital electronic medical records using PostgreSQL database queries. The collected information was organized into the following domains:

1. Demographic and clinical characteristics: patient age, gender, and National Institutes of Health Stroke Scale (NIHSS) score at admission were recorded (25).
2. Medical history and complications: pre-existing conditions and in-hospital complications were documented, including uremia, deep vein thrombosis (DVT), diabetes mellitus, hypertension, coronary artery disease, atrial fibrillation, cerebral herniation, hydrocephalus, hypoproteinemia, hyperuricemia, hyperlipidemia, and carotid artery stenosis.
3. Neuroimaging findings: brain involvement was assessed using computed tomography (CT) or magnetic resonance imaging (MRI). Cortical involvement was defined as lesions affecting the frontal, parietal, temporal, occipital, or insular lobes. Subcortical involvement included lesions in the basal ganglia, internal capsule, brainstem, cerebellum, periventricular region, centrum semiovale, and thalamus. For quantitative analysis, a cortical score was calculated as the number of affected lobes, and a subcortical score was calculated as the number of affected subcortical regions.
4. Vascular imaging: data from CT angiography (CTA), magnetic resonance angiography (MRA), or digital subtraction angiography (DSA) were used to identify stenosis or occlusion in major cerebral arteries, including the anterior cerebral artery, middle cerebral artery, posterior cerebral artery, vertebral artery, and basilar artery.
5. Laboratory measurements: venous blood samples were collected at admission following standard hospital protocols. The measured parameters included blood cell counts (platelet, WBC, and red blood cell counts); lipid profile [triglycerides (TG), high-density lipoprotein cholesterol (HDL-C), and low-density lipoprotein cholesterol (LDL-C)]; liver function tests [alanine aminotransferase (ALT), aspartate aminotransferase (AST), bilirubin, albumin]; renal function tests (urea, creatinine, uric acid); blood gas analysis [sodium (Na), potassium (K), chloride (Cl), calcium (Ca), phosphorus (P), lactate, anion gap, total carbon dioxide]; coagulation markers [international normalized ratio (INR), prothrombin time (PT), activated partial thromboplastin time (APTT), thrombin time (TT), D-dimer, fibrinogen]; cardiac enzymes [creatine kinase (CK), creatine kinase-MB isoenzyme (CK-MB), lactate dehydrogenase (LDH), α-hydroxybutyrate dehydrogenase (HBDH)]; and other markers including glycated hemoglobin (HbA1c) and C-reactive protein (CRP).

### 2.3 Statistical analysis

Baseline characteristics of the study participants were presented as means ± SD or medians (interquartile range) for continuous variables, as appropriate, and as numbers (percentages) for categorical variables. Comparisons across PWR quartiles were performed using one-way ANOVA (or the Kruskal–Wallis test for non-normally distributed data) for continuous variables and chi-square tests for categorical variables.

In the original study by Liu et al. (22), laboratory indicators with missing values exceeding 10% were excluded from analysis. For the remaining indicators with missing values of 10% or less, missing data were imputed using the random forest algorithm with default parameters. The imputation was performed iteratively, beginning with the variable with the fewest missing values; during each iteration, missing values in other features were temporarily replaced with 0, and the predicted values were inserted into the original feature matrix before proceeding to the next variable (22). This secondary analysis utilized the fully imputed dataset deposited in the Dryad repository (23); consequently, no additional missing data imputation or listwise deletion was required.

To evaluate the association between PWR and PSE incidence, multivariable logistic regression models were constructed with incremental adjustment: Model 1 was unadjusted; Model 2 adjusted for demographics (age, gender) and all comorbidities; Model 3 further adjusted for NIHSS score and cortical lobe involvement; and Model 4 was fully adjusted for all variables in Model 3 plus laboratory parameters (HbA1c, TG, LDL-C, ALT, Albumin, Creatinine, D-dimer, CK). Results were reported as odds ratios (ORs) with 95% confidence intervals (CIs). A trend test was performed across PWR quartiles by entering the median value of each quartile as a continuous variable in the models.

To explore the potential nonlinear relationship between PWR and PSE, we fitted a restricted cubic spline (RCS) regression adjusted for the same covariates as in Model 4. Additionally, a two-piecewise linear regression model was employed to explore potential nonlinearity in the PWR-PSE relationship, with the inflection point determined by a recursive algorithm that maximized model likelihood.

Subgroup analyses were conducted to evaluate the consistency of the association between PWR and PSE across various clinically relevant subgroups. These included deep vein thrombosis, diabetes, coronary artery disease, atrial fibrillation, basal ganglia involvement, paraventricular involvement, gender, common carotid artery plaque, internal carotid artery plaque, subcortical lobe involvement, anterior circulation involvement, and posterior circulation involvement. For each subgroup, the association between PWR and PSE was estimated using the fully adjusted model, and results were presented as ORs with 95%CIs. Interaction terms were introduced into the model to assess potential effect modification.

Finally, to assess the predictive ability of PWR compared to its individual components (platelet and WBC), receiver operating characteristic (ROC) curves were generated and the area under the curve (AUC) was calculated for each marker. Pairwise comparisons of AUCs were performed using DeLong’s test to determine whether the predictive performance of PWR was significantly superior to that of its individual components.

All statistical analyses were performed using SPSS (version 27.0; SPSS Inc., Chicago, IL) and R software (version 4.0.5). A two-tailed *p* value <0.05 was considered statistically significant.

## 3 Results

### 3.1 Baseline characteristics

A total of 21,459 patients with AIS were included in this study (Supplementary Figure S1). The baseline characteristics stratified by PWR quartiles are presented in Table 1. The mean age of the overall population was 66.41 ± 12.35 years, and 49.47% were male. Significant differences were observed across PWR quartiles for nearly all variables (*p* < 0.05 for most, except for basal ganglia, capsula interna, thalamus, and external carotid artery plaque involvement).

**Table 1:** Baseline characteristics stratified by PWR quartiles.

Variables	Total (*n* = 21,459)	Quartile 1 (*n* = 5,365)	Quartile 2 (*n* = 5,399)	Quartile 3 (*n* = 5,323)	Quartile 4 (*n* = 5,372)	*p*
Age (years)	66.41 ± 12.35	67.40 ± 14.48	68.92 ± 12.30	66.04 ± 11.26	63.28 ± 10.24	<0.001
Platelet (×10^9^/L)	189.90 ± 27.02	173.42 ± 26.79	183.99 ± 19.34	191.91 ± 18.23	210.31 ± 27.70	<0.001
WBC (×10^9^/L)	8.47 ± 1.59	10.02 ± 2.05	8.50 ± 0.91	7.94 ± 0.76	7.44 ± 0.82	<0.001
RBC (×10^9^/L)	4.31 ± 0.32	4.34 ± 0.37	4.32 ± 0.31	4.33 ± 0.30	4.27 ± 0.29	<0.001
HbA1c (%)	6.67 ± 0.92	6.67 ± 1.01	6.71 ± 0.85	6.62 ± 0.87	6.69 ± 0.96	<0.001
CRP (mg/L)	9.40 (4.70, 19.10)	18.60 (9.50, 36.90)	12.30 (6.90, 21.60)	7.20 (4.20, 12.70)	5.30 (3.30, 9.00)	<0.001
TG (mmol/L)	1.55 ± 0.43	1.42 ± 0.43	1.52 ± 0.41	1.57 ± 0.40	1.67 ± 0.46	<0.001
LDL-C (mmol/L)	2.69 ± 0.36	2.65 ± 0.40	2.67 ± 0.32	2.68 ± 0.32	2.75 ± 0.39	<0.001
HDL-C (mmol/L)	1.25 ± 0.15	1.27 ± 0.16	1.27 ± 0.15	1.25 ± 0.13	1.21 ± 0.14	<0.001
AST (U/L)	26.60 ± 13.40	33.12 ± 22.52	25.94 ± 8.80	23.99 ± 5.27	23.32 ± 6.70	<0.001
ALT (U/L)	24.38 ± 10.15	27.77 ± 16.17	24.45 ± 8.14	23.06 ± 5.71	22.24 ± 5.83	<0.001
Bilirubin (μmol/L)	15.26 ± 4.00	17.41 ± 4.78	15.68 ± 3.73	14.83 ± 2.87	13.11 ± 3.07	<0.001
Albumin (g/L)	40.91 ± 2.24	40.49 ± 2.52	40.53 ± 2.06	41.05 ± 1.96	41.59 ± 2.21	<0.001
Urea (mmol/L)	6.40 ± 1.43	6.48 ± 1.82	6.59 ± 1.34	6.44 ± 1.32	6.10 ± 1.11	<0.001
Creatinine (μmol/L)	75.50 (66.60, 86.60)	78.20 (70.30, 89.60)	77.60 (70.80, 87.60)	75.40 (66.50, 87.15)	68.30 (62.00, 82.80)	<0.001
Uric acid (μmol/L)	344.14 ± 58.84	344.12 ± 65.93	346.24 ± 55.27	351.39 ± 55.47	334.87 ± 56.85	<0.001
PT (s)	13.84 ± 1.14	13.92 ± 1.12	13.86 ± 1.23	13.87 ± 1.21	13.72 ± 0.96	<0.001
APTT (s)	35.66 ± 2.30	36.26 ± 2.58	35.82 ± 2.36	35.46 ± 2.02	35.08 ± 2.02	<0.001
TT (s)	16.44 ± 0.61	16.40 ± 0.76	16.51 ± 0.55	16.44 ± 0.59	16.42 ± 0.52	<0.001
INR (ratio)	1.08 ± 0.15	1.08 ± 0.14	1.08 ± 0.17	1.08 ± 0.16	1.06 ± 0.12	<0.001
D-dimer (ng/Ml)	0.92 (0.65, 1.49)	1.25 (0.78, 2.11)	1.04 (0.69, 1.78)	0.84 (0.63, 1.21)	0.78 (0.57, 1.06)	<0.001
Fibrinogen (g/L)	3.60 ± 0.47	3.66 ± 0.59	3.61 ± 0.44	3.54 ± 0.44	3.58 ± 0.41	<0.001
CK (U/L)	130.20 (103.40, 196.50)	207.00 (125.70, 470.40)	134.30 (107.05,186.80)	123.10 (102.10, 152.10)	112.60 (94.80,143.00)	<0.001
CK-MB (U/L)	12.60 (11.30, 15.20)	15.40 (12.10, 19.90)	12.90 (11.50, 15.00)	12.30 (11.40, 13.70)	11.60 (10.70, 13.00)	<0.001
LDH (U/L)	218.82 ± 79.01	253.57 ± 127.79	214.74 ± 57.76	205.55 ± 43.54	201.37 ± 41.21	<0.001
HBDH (U/L)	177.24 ± 49.21	195.29 ± 72.84	175.48 ± 41.13	169.39 ± 26.86	168.76 ± 38.72	<0.001
Na (mmol/L)	138.57 ± 1.76	137.93 ± 1.90	138.54 ± 1.45	138.68 ± 1.63	139.13 ± 1.80	<0.001
K (mmol/L)	3.79 ± 0.17	3.76 ± 0.21	3.80 ± 0.17	3.81 ± 0.13	3.78 ± 0.16	<0.001
Cl (mmol/L)	103.37 ± 1.97	102.45 ± 2.18	103.41 ± 1.59	103.48 ± 1.82	104.11 ± 1.88	<0.001
Ca (mmol/L)	2.20 ± 0.07	2.20 ± 0.08	2.20 ± 0.06	2.21 ± 0.06	2.21 ± 0.07	<0.001
P (mmol/L)	0.99 ± 0.09	0.98 ± 0.11	0.99 ± 0.08	0.99 ± 0.08	1.00 ± 0.09	<0.001
Lactate (mmol/L)	2.52 ± 0.41	2.59 ± 0.55	2.53 ± 0.37	2.49 ± 0.36	2.47 ± 0.33	<0.001
Anion gap (mmol/L)	12.38 ± 1.37	12.79 ± 1.82	12.30 ± 1.11	12.22 ± 1.04	12.20 ± 1.29	<0.001
Total carbon dioxide (mmol/L)	22.78 ± 1.22	22.68 ± 1.37	22.80 ± 1.11	22.89 ± 1.18	22.77 ± 1.18	<0.001
NIHSS	8.01 ± 2.94	9.22 ± 3.29	8.57 ± 2.90	7.82 ± 2.37	6.44 ± 2.33	<0.001
Gender, *n* (%)						<0.001
Male	10,616 (49.47)	2,201 (41.03)	2,409 (44.62)	2,455 (46.12)	3,551 (66.10)	
Female	10,843 (50.53)	3,164 (58.97)	2,990 (55.38)	2,868 (53.88)	1821 (33.90)	
Uremia (%)						<0.001
No	21,269 (99.11)	5,206 (97.04)	5,380 (99.65)	5,313 (99.81)	5,370 (99.96)	
Yes	190 (0.89)	159 (2.96)	19 (0.35)	10 (0.19)	2 (0.04)	
DVT (%)						<0.001
No	20,135 (93.83)	4,817 (89.79)	5,017 (92.92)	5,071 (95.27)	5,230 (97.36)	
Yes	1,324 (6.17)	548 (10.21)	382 (7.08)	252 (4.73)	142 (2.64)	
Fatty liver (%)						<0.001
No	17,214 (80.22)	4,565 (85.09)	4,501 (83.37)	4,312 (81.01)	3,836 (71.41)	
Yes	4,245 (19.78)	800 (14.91)	898 (16.63)	1,011 (18.99)	1,536 (28.59)	
Diabetes (%)						<0.001
No	14,137 (65.88)	3,404 (63.45)	3,385 (62.70)	3,783 (71.07)	3,565 (66.36)	
Yes	7,322 (34.12)	1961 (36.55)	2014 (37.30)	1,540 (28.93)	1807 (33.64)	
Hypertension (%)						<0.001
No	6,708 (31.26)	2,138 (39.85)	1743 (32.28)	1,691 (31.77)	1,136 (21.15)	
Yes	14,751 (68.74)	3,227 (60.15)	3,656 (67.72)	3,632 (68.23)	4,236 (78.85)	
Coronary artery disease (%)						<0.001
No	11,787 (54.93)	3,109 (57.95)	2,923 (54.14)	2,798 (52.56)	2,957 (55.04)	
Yes	9,672 (45.07)	2,256 (42.05)	2,476 (45.86)	2,525 (47.44)	2,415 (44.96)	
Atrial fibrillation (%)						<0.001
No	19,416 (90.48)	4,607 (85.87)	4,750 (87.98)	4,972 (93.41)	5,087 (94.69)	
Yes	2043 (9.52)	758 (14.13)	649 (12.02)	351 (6.59)	285 (5.31)	
Cerebral herniation (%)						<0.001
No	21,278 (99.16)	5,289 (98.58)	5,342 (98.94)	5,284 (99.27)	5,363 (99.83)	
Yes	181 (0.84)	76 (1.42)	57 (1.06)	39 (0.73)	9 (0.17)	
Hydrocephalus (%)						<0.001
No	21,178 (98.69)	5,241 (97.69)	5,317 (98.48)	5,272 (99.04)	5,348 (99.55)	
Yes	281 (1.31)	124 (2.31)	82 (1.52)	51 (0.96)	24 (0.45)	
Hyperuricemia (%)						<0.001
No	19,112 (89.06)	4,878 (90.92)	4,841 (89.66)	4,715 (88.58)	4,678 (87.08)	
Yes	2,347 (10.94)	487 (9.08)	558 (10.34)	608 (11.42)	694 (12.92)	
Hyperlipidemia (%)						<0.001
No	16,990 (79.17)	4,527 (84.38)	4,484 (83.05)	4,350 (81.72)	3,629 (67.55)	
Yes	4,469 (20.83)	838 (15.62)	915 (16.95)	973 (18.28)	1743 (32.45)	
Hypoproteinemia (%)						<0.001
No	19,038 (88.72)	4,221 (78.68)	4,605 (85.29)	4,969 (93.35)	5,243 (97.60)	
Yes	2,421 (11.28)	1,144 (21.32)	794 (14.71)	354 (6.65)	129 (2.40)	
Frontal lobe (%)						<0.001
No	20,540 (95.72)	5,026 (93.68)	5,128 (94.98)	5,165 (97.03)	5,221 (97.19)	
Yes	919 (4.28)	339 (6.32)	271 (5.02)	158 (2.97)	151 (2.81)	
Parietal lobe (%)						<0.001
No	20,796 (96.91)	5,118 (95.40)	5,207 (96.44)	5,206 (97.80)	5,265 (98.01)	
Yes	663 (3.09)	247 (4.60)	192 (3.56)	117 (2.20)	107 (1.99)	
Temporal lobe (%)						<0.001
No	20,823 (97.04)	5,125 (95.53)	5,189 (96.11)	5,215 (97.97)	5,294 (98.55)	
Yes	636 (2.96)	240 (4.47)	210 (3.89)	108 (2.03)	78 (1.45)	
Occipital lobe (%)						<0.001
No	21,064 (98.16)	5,196 (96.85)	5,283 (97.85)	5,263 (98.87)	5,322 (99.07)	
Yes	395 (1.84)	169 (3.15)	116 (2.15)	60 (1.13)	50 (0.93)	
Insular lobe (%)						<0.001
No	21,180 (98.70)	5,271 (98.25)	5,302 (98.20)	5,267 (98.95)	5,340 (99.40)	
Yes	279 (1.30)	94 (1.75)	97 (1.80)	56 (1.05)	32 (0.60)	
Basal ganglia (%)						0.088
No	20,491 (95.49)	5,119 (95.41)	5,133 (95.07)	5,114 (96.07)	5,125 (95.40)	
Yes	968 (4.51)	246 (4.59)	266 (4.93)	209 (3.93)	247 (4.60)	
Capsula interna (%)						0.508
No	21,445 (99.93)	5,363 (99.96)	5,394 (99.91)	5,321 (99.96)	5,367 (99.91)	
Yes	14 (0.07)	2 (0.04)	5 (0.09)	2 (0.04)	5 (0.09)	
Brainstem (%)						<0.001
No	21,187 (98.73)	5,268 (98.19)	5,323 (98.59)	5,274 (99.08)	5,322 (99.07)	
Yes	272 (1.27)	97 (1.81)	76 (1.41)	49 (0.92)	50 (0.93)	
Epencephalon (%)						<0.001
No	21,017 (97.94)	5,228 (97.45)	5,255 (97.33)	5,226 (98.18)	5,308 (98.81)	
Yes	442 (2.06)	137 (2.55)	144 (2.67)	97 (1.82)	64 (1.19)	
Paraventricular (%)						<0.001
No	20,412 (95.12)	5,161 (96.20)	5,203 (96.37)	5,097 (95.75)	4,951 (92.16)	
Yes	1,047 (4.88)	204 (3.80)	196 (3.63)	226 (4.25)	421 (7.84)	
Centrum semiovale (%)						<0.001
No	20,849 (97.16)	5,277 (98.36)	5,338 (98.87)	5,207 (97.82)	5,027 (93.58)	
Yes	610 (2.84)	88 (1.64)	61 (1.13)	116 (2.18)	345 (6.42)	
Thalamus (%)						0.643
No	21,222 (98.90)	5,303 (98.84)	5,345 (99.00)	5,268 (98.97)	5,306 (98.77)	
Yes	237 (1.10)	62 (1.16)	54 (1.00)	55 (1.03)	66 (1.23)	
Anterior cerebral artery (%)						<0.001
0	21,185 (98.72)	5,275 (98.32)	5,326 (98.65)	5,300 (99.57)	5,284 (98.36)	
1	274 (1.28)	90 (1.68)	73 (1.35)	23 (0.43)	88 (1.64)	
Middle cerebral artery (%)						<0.001
No	20,631 (96.14)	5,101 (95.08)	5,138 (95.17)	5,176 (97.24)	5,216 (97.10)	
Yes	828 (3.86)	264 (4.92)	261 (4.83)	147 (2.76)	156 (2.90)	
Posterior cerebral artery (%)						0.002
No	21,397 (99.71)	5,358 (99.87)	5,389 (99.81)	5,304 (99.64)	5,346 (99.52)	
Yes	62 (0.29)	7 (0.13)	10 (0.19)	19 (0.36)	26 (0.48)	
Vertebral artery (%)						0.004
No	20,807 (96.96)	5,188 (96.70)	5,210 (96.50)	5,164 (97.01)	5,245 (97.64)	
Yes	652 (3.04)	177 (3.30)	189 (3.50)	159 (2.99)	127 (2.36)	
Basilar artery (%)						<0.001
No	21,268 (99.11)	5,309 (98.96)	5,333 (98.78)	5,279 (99.17)	5,347 (99.53)	
Yes	191 (0.89)	56 (1.04)	66 (1.22)	44 (0.83)	25 (0.47)	
Common carotid artery plaque (%)						<0.001
No	16,636 (77.52)	4,341 (80.91)	4,115 (76.22)	4,299 (80.76)	3,881 (72.24)	
Yes	4,823 (22.48)	1,024 (19.09)	1,284 (23.78)	1,024 (19.24)	1,491 (27.76)	
Internal carotid artery plaque (%)						<0.001
No	19,997 (93.19)	5,048 (94.09)	5,051 (93.55)	5,014 (94.20)	4,884 (90.92)	
Yes	1,462 (6.81)	317 (5.91)	348 (6.45)	309 (5.80)	488 (9.08)	
External carotid artery plaque (%)						0.276
No	21,258 (99.06)	5,326 (99.27)	5,346 (99.02)	5,272 (99.04)	5,314 (98.92)	
Yes	201 (0.94)	39 (0.73)	53 (0.98)	51 (0.96)	58 (1.08)	
Subcortical lobe (%)						<0.001
No	19,016 (88.62)	4,756 (88.65)	4,773 (88.41)	4,806 (90.29)	4,681 (87.14)	
Yes	2,443 (11.38)	609 (11.35)	626 (11.59)	517 (9.71)	691 (12.86)	
Anterior circulation (%)						<0.001
No	20,046 (93.42)	4,945 (92.17)	5,014 (92.87)	5,064 (95.13)	5,023 (93.50)	
Yes	1,413 (6.58)	420 (7.83)	385 (7.13)	259 (4.87)	349 (6.50)	
Posterior circulation (%)						<0.001
No	16,958 (79.03)	4,345 (80.99)	4,401 (81.52)	4,299 (80.76)	3,913 (72.84)	
Yes	4,501 (20.97)	1,020 (19.01)	998 (18.48)	1,024 (19.24)	1,459 (27.16)	
Post-stroke epilepsy (%)						<0.001
No	20,523 (95.64)	4,612 (85.96)	5,263 (97.48)	5,290 (99.38)	5,358 (99.74)	
Yes	936 (4.36)	753 (14.04)	136 (2.52)	33 (0.62)	14 (0.26)	

Continuous variables were described by mean ± standard deviation or median (interquartile range) and categorical variables were presented by number (percentage).

PWR, platelet to white blood cell ratio; WBC, white blood cell; RBC, red blood cell; HbA1c, glycated hemoglobin; CRP, C-reactive protein; TG, triglyceride; LDL-c, low-density lipoprotein cholesterol; HDL-c, high-density lipoprotein cholesterol; AST, aspartate aminotransferase; ALT, alanine aminotransferase; PT, prothrombin time; APTT, activated partial thromboplastin time; TT, thrombin time; INR, international normalized ratio; CK, creatine kinase; CK-MB, creatine kinase-MB isoenzyme; LDH, lactate dehydrogenase; HBDH, hydroxybutyrate dehydrogenase; Na, sodium; K, potassium; Cl, chloride; Ca, calcium; P, phosphorus; NIHSS, National Institutes of Health Stroke Scale; DVT, deep vein thrombosis.

Patients in the highest PWR quartile (Q4) were younger (63.28 ± 10.24 vs. 67.40 ± 14.48 years in Q1) and predominantly male (66.10% vs. 41.03% in Q1). Stroke severity showed a clear inverse gradient, with NIHSS scores decreasing progressively from 9.22 ± 3.29 in Q1 to 6.44 ± 2.33 in Q4. Conditions associated with inflammation and coagulation, including deep vein thrombosis (10.21% in Q1 vs. 2.64% in Q4), atrial fibrillation (14.13% vs. 5.31%), and uremia (2.96% vs. 0.04%), were more prevalent in lower PWR quartiles. Conversely, metabolic conditions such as fatty liver, hypertension, and hyperlipidemia were more common in Q4. Cortical involvement across all lobes was significantly more frequent in lower quartiles (*p* < 0.001). For example, frontal lobe involvement decreased from 6.32% in Q1 to 2.81% in Q4. In contrast, paraventricular and centrum semiovale involvement were more prevalent in Q4 (7.84 and 6.42%, respectively), suggesting potential differences in lesion distribution. Middle cerebral artery stenosis or occlusion was more common in lower quartiles (4.92% in Q1 vs. 2.90% in Q4), while common and internal carotid artery plaques were more prevalent in Q4, consistent with the higher metabolic comorbidity burden in this group. Inflammatory and coagulation markers showed striking gradients. CRP decreased markedly from 18.60 mg/L in Q1 to 5.30 mg/L in Q4. D-dimer followed a similar pattern (1.25 vs. 0.78 ng/mL). Cardiac enzymes, including CK and LDH, were markedly elevated in Q1, suggesting more extensive tissue damage. Lipid parameters (TG, LDL-C) showed modest increases in higher quartiles, consistent with the metabolic profile of Q4.

The overall incidence of PSE within 1 year post-stroke was 4.36% (936/21,459) in the total cohort. When stratified by PWR quartiles, the absolute risk of PSE decreased progressively from 14.04% (753/5,365) in the lowest quartile (Q1) to 2.52% (136/5,399) in Q2, 0.62% (33/5,323) in Q3, and 0.26% (14/5,372) in the highest quartile (Q4). Supplementary Table S1 displays baseline characteristics according to PSE status.

### 3.2 Association between PWR and PSE

Table 2 presents the results of the logistic regression analyses. In the unadjusted Model 1, higher PWR was significantly associated with a lower incidence of PSE (OR per 1-SD increase: 0.158; 95% CI: 0.145–0.173; *p* < 0.001). This inverse association remained statistically significant across all levels of adjustment.

**Table 2:** Association between PWR and PSE incidence in patients with ischemic stroke.

Characteristics	Model 1		Model 2		Model 3		Model 4	
	OR (95%CI)	*p*	OR (95%CI)	*p*	OR (95%CI)	*p*	OR (95%CI)	*p*
PWR (per 1 SD)	0.158 (0.145–0.173)	<0.001	0.122 (0.109–0.135)	<0.001	0.145 (0.130–0.162)	<0.001	0.214 (0.186–0.245)	<0.001
PWR (quartile)								
Quartile 1	Ref		Ref		Ref		Ref	
Quartile 2	0.158 (0.131–0.191)	<0.001	0.151 (0.125–0.182)	<0.001	0.170 (0.139–0.208)	<0.001	0.264 (0.211–0.330)	<0.001
Quartile 3	0.038 (0.027–0.054)	<0.001	0.035 (0.024–0.049)	<0.001	0.049 (0.034–0.071)	<0.001	0.093 (0.064–0.136)	<0.001
Quartile 4	0.016 (0.009–0.027)	<0.001	0.014 (0.008–0.023)	<0.001	0.025 (0.014–0.044)	<0.001	0.033 (0.018–0.059)	<0.001
*p* for trend		<0.001		<0.001		<0.001		<0.001

Model 1: unadjusted.

Model 2: gender, age, comorbidities and complications (uremia, deep vein thrombosis, fatty liver, diabetes, hypertension, coronary artery disease, atrial fibrillation, cerebral herniation, hydrocephalus, hypoproteinemia, hyperuricemia, hyperlipidemia, and carotid stenosis).

Model 3: adjusted for variables in Model 2 plus NIHSS, and cortical lobes (frontal, parietal, temporal, occipital, and insular).

Model 4: adjusted for variables in Model 3 plus HbA1c, TG, LDL-C, ALT, albumin, creatinine, D-dimer and CK.

PWR, platelet to white blood cell ratio; PSE, post-stroke epilepsy; OR, odds ratio; CI, confidence interval; Ref., reference; HbA1c, glycated hemoglobin; TG, triglyceride; LDL-C, low-density lipoprotein cholesterol; ALT, alanine aminotransferase; CK, creatine kinase.

When PWR was analyzed in quartiles, a clear dose–response relationship was observed. Compared to patients in the lowest quartile (Q1), the risk of PSE decreased progressively for those in higher quartiles. In the fully adjusted Model 4, the ORs (95% CI) for Q2, Q3, and Q4 were 0.264 (0.211–0.330), 0.093 (0.064–0.136), and 0.033 (0.018–0.059), respectively (all *p* < 0.001). The P for trend across the quartiles was highly significant (*p* < 0.001) in all models, further confirming the inverse dose–response relationship.

### 3.3 Nonlinear relationship

**Figure 1:**
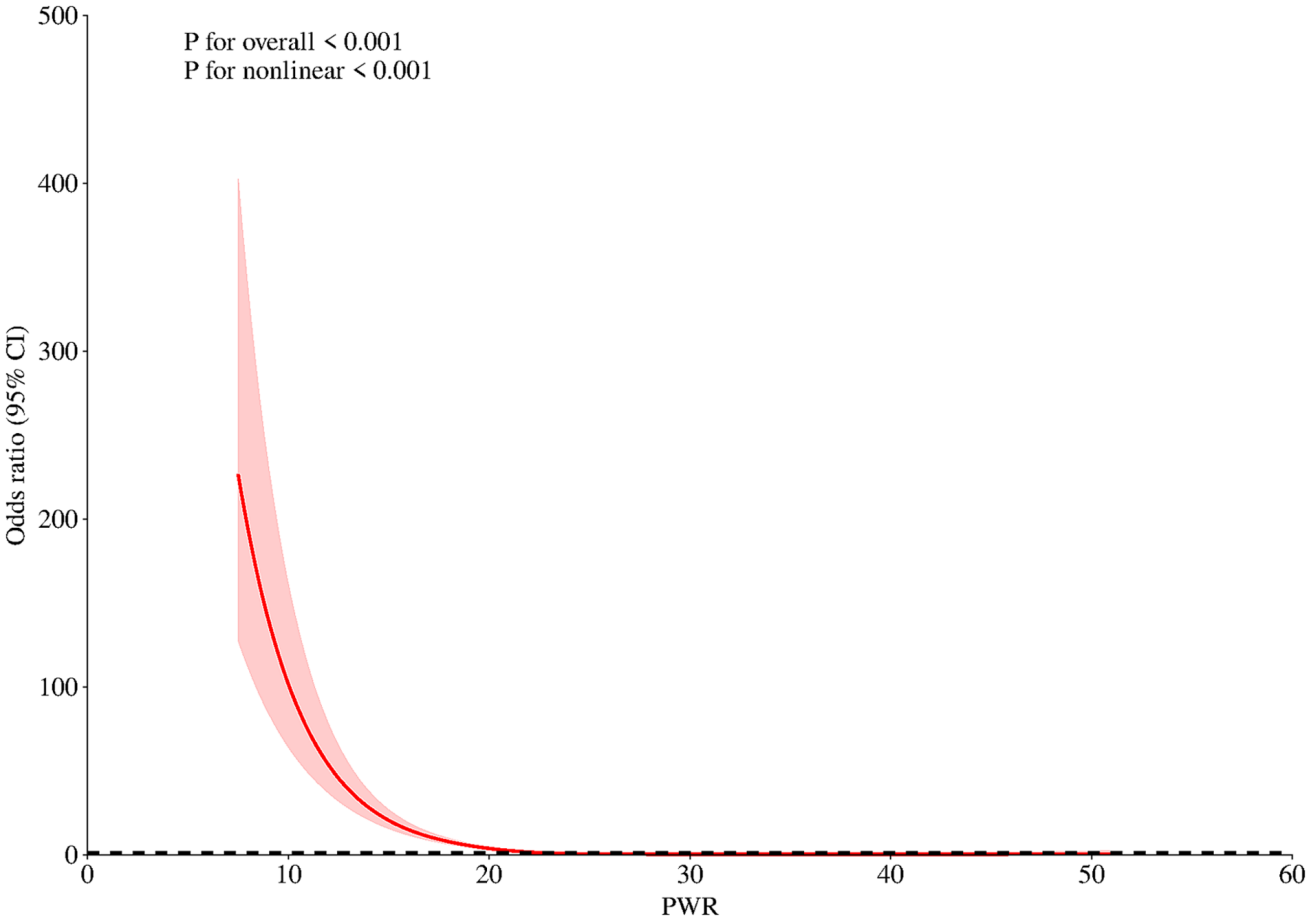
Restricted cubic spline model for the association between platelet to white blood cell ratio (PWR) and post-stroke epilepsy (PSE) incidence in patients with ischemic stroke. The solid red line represents the adjusted odds ratio (OR) for PSE incidence across the continuum of PWR values, with the reference point set at the median PWR value. The pink shaded area represents the 95% confidence interval. The horizontal dotted line indicates the null effect (OR = 1). *p* for overall association <0.001; *p* for nonlinearity <0.001.

The restricted cubic spline analysis, adjusted for all covariates in Model 4, revealed a significant nonlinear relationship between PWR and PSE risk (Figure 1). The *p* for overall association was <0.001, and the *p* for nonlinearity was also <0.001. The curve showed that the risk of PSE decreased steeply with increasing PWR at lower values, with a gradual flattening of the slope at higher PWR levels.

To further characterize this nonlinear relationship, a two-piecewise linear regression model was employed. The log-likelihood ratio test confirmed that the piecewise model fit the data significantly better than a standard linear model (*p* for likelihood ratio test <0.001) (Table 3). This analysis identified an inflection point at a PWR value of approximately 20.3. Below this inflection point, each unit increase in PWR was associated with a 28% reduction in PSE risk (OR: 0.72; 95% CI: 0.69–0.76; *p* < 0.001); above this point, each unit increase was associated with a 30% reduction (OR: 0.70; 95% CI: 0.64–0.76; *p* < 0.001). Although the magnitude of risk reduction was similar on both sides of this inflection point, the statistically significant improvement in model fit (likelihood ratio test *p* < 0.001) supports the presence of a nonlinear relationship with a change in slope at PWR 20.3, rather than a simple linear association across the entire range.

**Table 3:** Two-piecewise logistic regression analysis of PWR on PSE in patients with ischemic stroke using a two-piecewise logistic regression.

Model	Adjusted OR (95% CI)	*p*
Model 1 Fitting model by standard linear regression	0.70 (0.68–0.73)	<0.001
Model 2 Fitting model by two-piecewise linear regression		
Inflection point	20.31	
<20.31	0.72 (0.69–0.76)	<0.001
≥20.31	0.70 (0.64–0.76)	<0.001
*p* for likelihood test		<0.001

PWR, platelet to white blood cell ratio; PSE, post-stroke epilepsy; OR, odds ratio; CI, confidence interval.

### 3.4 Subgroup analyses

**Figure 2:**
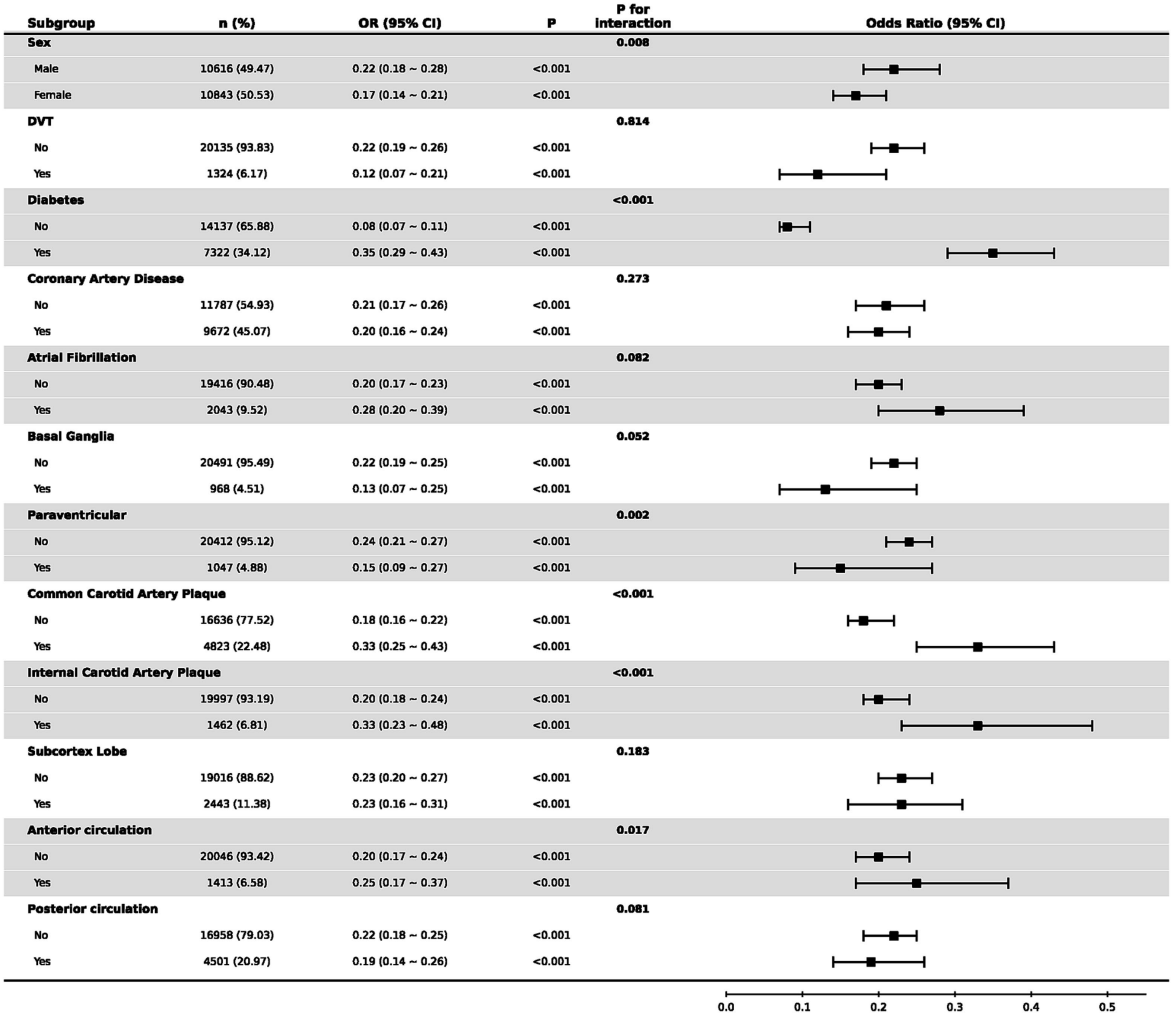
Subgroup analyses for association between PWR (per 1 SD) and PSE in patients with ischemic stroke. PWR, platelet to white blood cell ratio; PSE, post-stroke epilepsy; OR, odds ratio; CI, confidence interval.

Subgroup analyses were conducted to evaluate the consistency of the inverse association between PWR and PSE across various clinically relevant populations (Figure 2). The protective effect of PWR was consistently observed across all subgroups examined, including presence or absence of deep vein thrombosis, diabetes, coronary artery disease, atrial fibrillation, basal ganglia involvement, paraventricular involvement, gender, common carotid artery plaque, internal carotid artery plaque, subcortical lobe involvement, anterior circulation involvement, and posterior circulation involvement.

Significant effect modification was observed for several subgroups. The association between PWR and PSE was significantly stronger in patients without diabetes (OR: 0.08; 95% CI: 0.07–0.11) compared to those with diabetes (OR: 0.35; 95% CI: 0.29–0.43) (*p* for interaction <0.001). Similarly, the protective effect was more pronounced in patients without common carotid artery plaque (OR: 0.18; 95% CI: 0.16–0.22) than in those with plaque (OR: 0.33; 95% CI: 0.25–0.43) (*p* for interaction <0.001), and in patients without internal carotid artery plaque (OR: 0.20; 95% CI: 0.18–0.24) compared to those with plaque (OR: 0.33; 95% CI: 0.23–0.48) (*p* for interaction <0.001). Significant interactions were also observed for paraventricular involvement (*p* for interaction = 0.002), gender (*p* for interaction = 0.008), and anterior circulation involvement (*p* for interaction = 0.017). No significant effect modification was found for coronary artery disease, atrial fibrillation, basal ganglia involvement, subcortical lobe involvement, or posterior circulation involvement (all *p* for interaction >0.05).

### 3.5 Predictive performance of PWR

**Figure 3:**
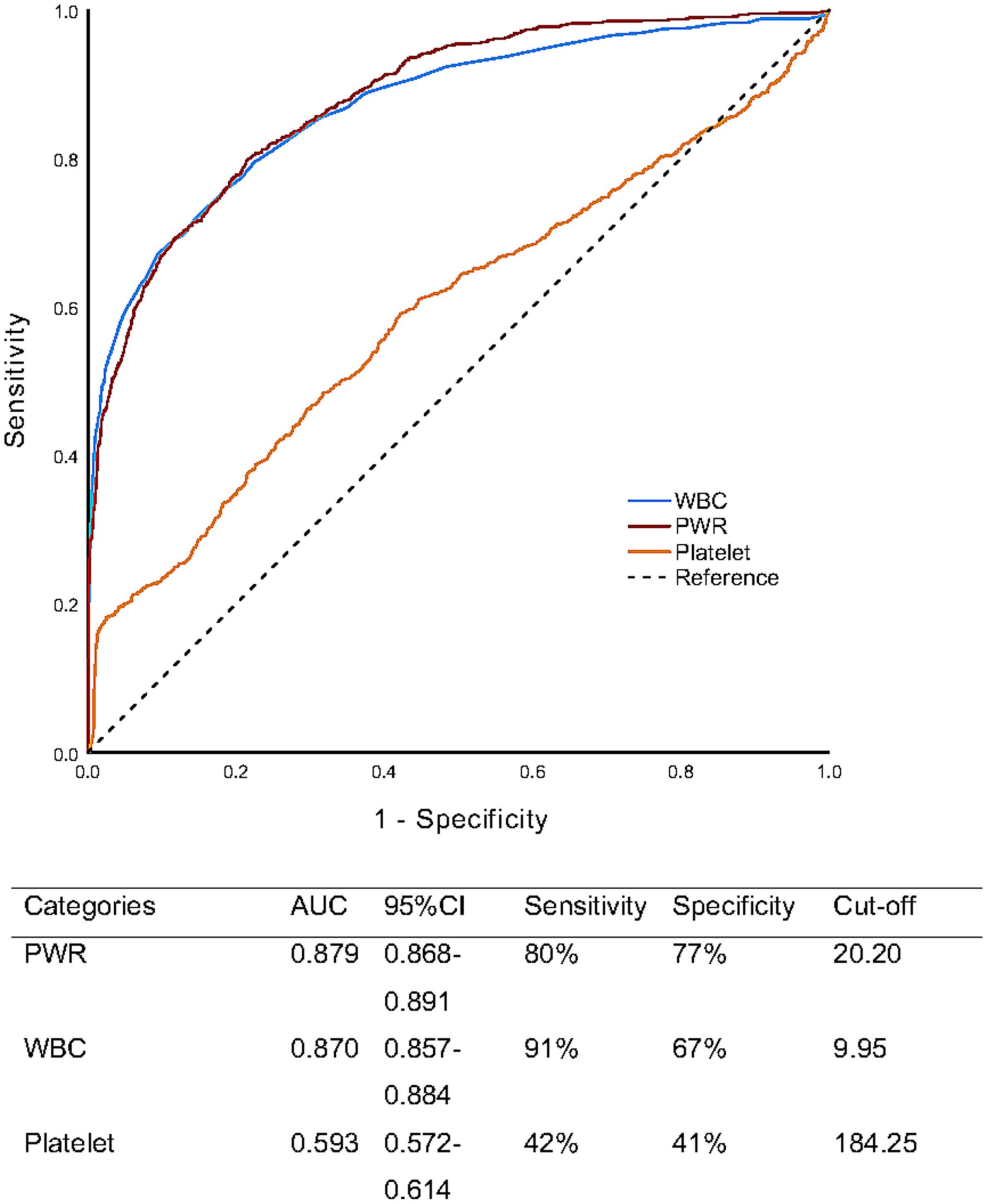
Receiver operating characteristic analysis comparing the predictive ability of platelet to white blood cell ratio (PWR), white blood cell (WBC), and platelet for post-stroke epilepsy in patients with ischemic stroke.

The ROC curve analysis compared the predictive ability of PWR with its individual components (platelet and WBC). PWR demonstrated good predictive capacity for PSE, with an AUC of 0.879 (95% CI, 0.868–0.891) (Figure 3, Table 4). Pairwise comparisons using DeLong’s test confirmed that the predictive performance of PWR was significantly better than both platelet count (*p* < 0.001) and WBC (*p* = 0.036). These findings suggested that the combination of these two parameters into a single ratio provides incremental predictive value over either component alone. To further illustrate the clinical utility of PWR, we identified the optimal cut-off value for predicting PSE using Youden’s index from the ROC analysis, which was 20.20 (Figure 3).

**Table 4:** Paired comparison of ROC curves (DeLong’s test).

Categories	Difference of AUC	Standard error	95%CI	*z*-value	*p*
PWR vs. Platelet	0.287	0.130	0.264–0.309	25.321	<0.001
PWR vs. WBC	0.009	0.113	0.001–0.018	2.099	0.036

ROC, receiver operating characteristic; AUC, area under curve; CI, confidence interval; PWR, platelet to white blood cell ratio; WBC, white blood cell.

## 4 Discussion

In this large multicenter retrospective cohort study of 21,459 patients with AIS, we investigated the association between the PWR and the incidence of PSE. Our main findings were summarized as follows: (1) Higher PWR was independently and significantly associated with a lower risk of PSE, with a clear dose–response relationship across quartiles; (2) This inverse association persisted after comprehensive adjustment for demographic characteristics, comorbidities, neuroimaging findings, and laboratory parameters; (3) A nonlinear relationship was identified, with a change in slope at a PWR value of 20.31; the protective effect of PWR against PSE was consistently significant both below (OR: 0.72) and above (OR: 0.70) this point; (4) Subgroup analyses revealed that the protective effect was consistent across most clinically relevant populations, with significant interactions observed for diabetes, carotid artery plaque, paraventricular involvement, gender, and anterior circulation involvement; and (5) PWR demonstrated superior predictive performance for PSE compared to its individual components (platelet and WBC alone), as confirmed by DeLong’s test.

To the best of our knowledge, this is the first study to specifically investigate the association between PWR and PSE. While a recent study examined systemic inflammatory markers in relation to PSE using competing risk analysis (9), no previous research has focused on the PWR as a composite marker in this context. Our findings therefore provided novel evidence that the integration of platelet and WBC into a single ratio may offer unique prognostic information for PSE risk stratification.

The relationship between PWR and stroke outcomes has been explored in several previous studies, though none have extended this to epilepsy. Recent studies have demonstrated the prognostic value of PWR in various acute ischemic stroke populations (13–15). For instance, one study reported that PWR was associated with mechanical thrombectomy outcomes in patients with acute ischemic stroke (13). Another investigation found that PWR, in combination with NIHSS score, had clinical significance in predicting stroke severity and outcomes (14). Additionally, PWR has been identified as a prognostic predictor for 90-day functional outcomes in ischemic stroke patients receiving intravenous thrombolysis (15). These studies suggested that PWR might reflect the balance between pro-inflammatory and pro-thrombotic pathways, with a lower ratio indicating a more unfavorable profile. Our findings extend this concept to post-stroke epileptogenesis, demonstrating that the prognostic value of PWR may encompass not only acute stroke outcomes and treatment responses but also long-term neurological sequelae such as epilepsy.

Regarding the individual components of PWR, both platelet and WBC have been independently associated with stroke outcomes and epilepsy risk in previous literature, although studies specifically on PSE remain limited. Elevated WBC, as a marker of systemic inflammation, has been associated with poorer outcomes after stroke (16–18), including increased risk of seizures (19). The underlying mechanism may involve inflammation-induced neuronal hyperexcitability and disruption of the blood–brain barrier (26–28). Conversely, platelets play a complex role in stroke pathophysiology. While excessive platelet activation contributes to thrombosis and ischemic injury (29), platelets also contain and release various neurotrophic factors that may promote neuronal survival and tissue repair (20, 21). Studies on epilepsy (not specifically post-stroke) have suggested that platelet count may be involved in seizure generation, though findings have been inconsistent (30–32). The PWR, by integrating these two opposing pathways, may provide a more comprehensive assessment of the inflammatory-thrombotic balance than either marker alone, which is supported by our ROC analysis showing superior predictive performance of the combined ratio.

Several biological mechanisms may explain the protective association between higher PWR and lower PSE risk. First, from an inflammatory perspective, a higher PWR reflects relatively lower WBC count, indicating a less pronounced systemic inflammatory response. Neuroinflammation is a well-established contributor to epileptogenesis following brain injury (33, 34). Activated leukocytes release pro-inflammatory cytokines such as interleukin-1β and tumor necrosis factor-α, which can enhance neuronal excitability, reduce seizure threshold, and promote the development of epileptic networks (34, 35). The striking gradient in C-reactive protein and D-dimer levels across PWR quartiles observed in our study supports this inflammatory hypothesis, as both markers decreased progressively from the lowest to the highest PWR quartile. Second, platelets may exert direct neuroprotective effects. Beyond their traditional role in hemostasis, platelets contain and release various growth factors, including platelet-derived growth factor (PDGF), vascular endothelial growth factor (VEGF), and brain-derived neurotrophic factor (20, 36). These factors promote angiogenesis, neurogenesis, and synaptic plasticity, which may facilitate recovery after stroke and reduce the likelihood of epileptogenesis (37). Third, the PWR may reflect the integrity of the blood–brain barrier (BBB). Both inflammation and platelet dysfunction can compromise BBB integrity (38–40). BBB disruption allows extravasation of serum proteins, particularly albumin, into the brain parenchyma, where they can bind to astrocytes and induce downregulation of potassium channels and impaired glutamate uptake, leading to neuronal hyperexcitability and seizures (41, 42). The association between PWR and PSE may therefore be mediated, in part, through effects on BBB stability.

The nonlinear relationship between PWR and PSE risk warrants careful interpretation. The restricted cubic spline analysis revealed a significant overall nonlinear association (*p* for nonlinearity <0.001), with a visual trend suggesting that the protective effect of PWR may attenuate at higher values—a pattern consistent with a “diminishing returns” phenomenon. To further characterize this nonlinearity, we applied a two-piecewise linear regression model, which detected a statistically significant change in slope at an inflection point of 20.31 (*p* for likelihood ratio test <0.001). However, the nearly identical effect estimates below (OR: 0.72) and above (OR: 0.70) this inflection point indicate that the magnitude of this slope change is numerically modest. Therefore, the most clinically relevant message from these complementary analyses is that the inverse association between PWR and PSE is consistent across the entire observed range of PWR, without evidence that the protective effect is lost or substantially diminished at higher levels. The inflection point at 20.31 should be viewed as a statistical descriptor of the relationship’s shape rather than a validated clinical decision threshold. Future prospective studies with larger sample sizes and serial PWR measurements are needed to determine whether this inflection point has practical utility for risk stratification or guiding anti-inflammatory/thrombotic management strategies.

The subgroup analyses revealed several noteworthy interactions. The protective effect of PWR was significantly stronger in patients without diabetes compared to those with diabetes. Diabetes is associated with chronic low-grade inflammation, endothelial dysfunction, and microvascular complications, which may attenuate the beneficial effects of a favorable inflammatory-thrombotic profile (43, 44). Similarly, the presence of carotid artery plaque modified the association, with a weaker protective effect observed in patients with plaque. This may reflect the contribution of large-artery atherosclerosis to stroke pathogenesis and subsequent epilepsy risk, potentially overwhelming the protective effects of higher PWR (45, 46). The significant interaction with gender, showing a stronger protective effect in females, warrants further investigation. Gender differences in inflammatory responses and platelet function have been documented, with estrogen exerting complex effects on both systems (47, 48). The stronger association in women may have implications for personalized risk assessment and prevention strategies. Interestingly, paraventricular and anterior circulation involvement also showed significant interactions. The risk of PSE varies according to the specific brain regions involved. These findings may reflect differences in the inflammatory response or blood–brain barrier integrity across different brain regions, which could interact with systemic inflammatory markers such as PWR. Given the exploratory nature of these subgroup analyses and the lack of previous studies on this topic, our findings should be interpreted with caution and warrant confirmation in future prospective investigations.

The superior predictive performance of PWR compared to its individual components, as demonstrated by ROC curve analysis and confirmed by DeLong’s test, supports the clinical utility of this composite marker. The AUC of 0.879 indicates good discriminative ability for identifying patients at risk of developing PSE. This finding is consistent with the broader literature on composite inflammatory ratios, which often outperform individual markers due to their ability to capture complex biological interactions (9, 49). From a practical perspective, PWR is easily calculable from routine complete blood counts, requires no additional cost or specialized equipment, and could be readily integrated into clinical practice for early risk stratification. Given the paucity of research on PWR in the context of PSE, our study provides an important foundation for future investigations.

### 4.1 Limitations

Several limitations of this study should be acknowledged. First, as a retrospective observational study, we cannot establish causality, and residual confounding by unmeasured variables cannot be excluded. Although we adjusted for a comprehensive set of covariates, factors such as medication use (e.g., antiplatelet agents, statins) and stroke etiology (e.g., cardioembolic vs. large artery atherosclerosis) were not available in the dataset and may have influenced the observed associations. Second, and particularly important, individual-level data regarding antithrombotic therapies—including antiplatelet agents (e.g., aspirin, clopidogrel) and anticoagulants—were not available in the dataset. These medications were highly relevant to our study because they may directly influence platelet count, platelet volume, platelet function, and the systemic inflammatory-thrombotic milieu, thereby potentially confounding the baseline PWR value. However, several considerations may partially mitigate this concern. Antithrombotic therapy represents the standard of care for acute ischemic stroke in China, and the participating centers followed national guideline-concordant protocols, which likely minimized between-patient heterogeneity in exposure. In addition, antiplatelet agents primarily affect platelet activation and aggregation rather than circulating platelet count in a consistent, dose-dependent manner. To the extent that antiplatelet therapy does reduce platelet count, this would tend to lower PWR and bias the observed association toward the null, suggesting that our reported protective effect of higher PWR may, if anything, be conservative. Furthermore, the WBC component of PWR is less directly affected by antithrombotic agents than the platelet component. Nevertheless, we cannot exclude the possibility that unmeasured antithrombotic use influenced our results, and we strongly recommend that future prospective studies collect detailed, time-stamped medication data to further clarify this relationship. Third, the diagnosis of PSE relied on clinical documentation, and subclinical or unreported seizures may have been missed, potentially leading to underestimation of the true incidence. Fourthly, direct infarct volume measurements were not available in the Dryad dataset. Infarct size is a well-established predictor of PSE risk, and its absence represents a potential limitation. In our analysis, we adjusted for NIHSS score and cortical/subcortical involvement scores as proxies for stroke severity and lesion burden; however, these clinical and topographic measures do not fully substitute for precise volumetric quantification. Future studies incorporating automated infarct volume segmentation are warranted to determine whether the prognostic value of PWR is independent of, or synergistic with, lesion size.

## 5 Conclusion

This multicenter study provided the first evidence that higher PWR was independently associated with lower PSE risk in AIS patients. The inverse relationship showed a dose–response pattern and remained robust after comprehensive adjustment. PWR demonstrated superior predictive performance over its individual components. Prospective studies are warranted to validate our findings and elucidate underlying mechanisms.

## Data Availability

The datasets presented in this study can be found in online repositories. The names of the repository/repositories and accession number(s) can be found at: https://datadryad.org/dataset/doi:10.5061/dryad.w0vt4b92c.

## Footnotes

1
https://datadryad.org
