# Supplementary material for: Association between platelet to white blood cell ratio and post-stroke epilepsy in patients with acute ischemic stroke: a multicenter retrospective cohort study
Source: Front Neurol. 2026 Jul 29;17:1870448. doi: 10.3389/fneur.2026.1870448 (PMC13462387; doi:10.3389/fneur.2026.1870448)
Supplement: Supplementary file 1 [file Table_1.DOCX]

**Supplementary Figure 1
Patient Selection Flow Diagram**

**STEP 1: INITIAL DATABASE QUERY**

**42,079**

records retrieved from the stroke database

Four hospitals in Chongqing, China

▼

| **STEP 2: FIRST-LEVEL SCREENING (Stroke Type & Data Completeness)** | | | |
| --- | --- | --- | --- |
| **Excluded:** | Hemorrhagic strokes | | 4,565 |
|  | Prior stroke history | | 2,154 |
|  | Transient ischemic attack (TIA) | | 3,570 |
|  | Stroke of unclear etiology | | 561 |
|  | Missing essential data | | 6,496 |
|  | **Subtotal excluded** | | **17,346** |
| **Included after Step 2: Ischemic or lacunar stroke with new onset** | | **24,733** | |

▼

| **STEP 3: SECOND-LEVEL SCREENING (Epilepsy-Related Factors & In-Hospital Death)** | | | |
| --- | --- | --- | --- |
| **Excluded:** | Seizures attributable to other factors (brain tumors, intracranial vascular malformations, traumatic brain injury) | | 865 |
|  | History of seizures | | 152 |
|  | Death during hospitalization | | 1,444 |
|  | **Subtotal excluded** | | **2,461** |
| **Included after Step 3: Eligible patients remaining** | | **22,272** | |

▼

| **STEP 4: THIRD-LEVEL SCREENING (Follow-up Status)** | | | |
| --- | --- | --- | --- |
| **Excluded:** | Lost to follow-up (no outpatient records or unreachable by telephone) OR death within 3 months of stroke incident | | **813** |
| **Included after Step 4: Patients with complete follow-up** | | **21,459** | |

▼

**FINAL ANALYTICAL COHORT**

**21,459 patients included**

▼	▼

| **PSE POSITIVE** |  | **PSE NEGATIVE** |
| --- | --- | --- |
| **936** |  | **20,523** |
| (4.36%) |  | (95.64%) |
| Post-stroke epilepsy within 1 year |  | No post-stroke epilepsy within 1 year |

Table S1 Baseline characteristics according to PSE status.

| Variables | Total  (n = 21459) | Non-PSE  (n = 5365) | PSE  (n = 5399) | P |
| --- | --- | --- | --- | --- |
|  |  |  |  |  |
| Age (years) | 66.41 ± 12.35 | 66.48 ± 12.29 | 64.90 ± 13.45 | <0.001 |
| Platelet (×109/L) | 189.90 ± 27.02 | 190.31 ± 26.42 | 180.92 ± 36.76 | <0.001 |
| WBC (×109/L) | 8.47 ± 1.59 | 8.32 ± 1.29 | 11.84 ± 3.07 | <0.001 |
| RBC (×109/L) | 4.31 ± 0.32 | 4.31 ± 0.32 | 4.42 ± 0.27 | <0.001 |
| HbA1c (%) | 6.67 ± 0.92 | 6.67 ± 0.92 | 6.77 ± 1.05 | 0.002 |
| CRP (mg/L) | 9.40 (4.70, 19.10) | 8.90 (4.50, 17.60) | 33.50 (20.88, 88.62) | <0.001 |
| TG (mmol/L) | 1.55 ± 0.43 | 1.54 ± 0.43 | 1.67 ± 0.48 | <0.001 |
| LDL-C (mmol/L) | 2.69 ± 0.36 | 2.69 ± 0.36 | 2.69 ± 0.37 | 0.829 |
| HDL-C (mmol/L) | 1.25 ± 0.15 | 1.25 ± 0.15 | 1.24 ± 0.14 | 0.342 |
| AST (U/L) | 26.60 ± 13.40 | 26.06 ± 12.87 | 38.43 ± 18.31 | <0.001 |
| ALT (U/L) | 24.38 ± 10.15 | 24.26 ± 10.13 | 26.95 ± 10.38 | <0.001 |
| Bilirubin (µmol/L) | 15.26 ± 4.00 | 15.20 ± 3.99 | 16.53 ± 4.03 | <0.001 |
| Albumin (g/L) | 40.91 ± 2.24 | 40.92 ± 2.24 | 40.77 ± 2.32 | 0.053 |
| Urea (mmol/L) | 6.40 ± 1.43 | 6.41 ± 1.44 | 6.32 ± 1.28 | 0.065 |
| Creatinine (µmol/L) | 75.50 (66.60, 86.60) | 75.30 (66.40, 86.50) | 77.60 (71.20, 89.90) | <0.001 |
| Uric acid (µmol/L) | 344.14 ± 58.84 | 344.19 ± 58.05 | 343.01 ± 74.22 | 0.632 |
| PT (s) | 13.84 ± 1.14 | 13.84 ± 1.16 | 13.82 ± 0.63 | 0.242 |
| APTT (s) | 35.66 ± 2.30 | 35.68 ± 2.31 | 35.04 ± 1.89 | <0.001 |
| TT (s) | 16.44 ± 0.61 | 16.43 ± 0.61 | 16.61 ± 0.55 | <0.001 |
| INR (ratio) | 1.08 ± 0.15 | 1.08 ± 0.15 | 1.07 ± 0.07 | <0.001 |
| D-dimer (ng/Ml) | 0.92 (0.65, 1.49) | 0.89 (0.65, 1.42) | 1.99 (1.38, 7.24) | <0.001 |
| Fibrinogen (g/L) | 3.60 ± 0.47 | 3.60 ± 0.46 | 3.52 ± 0.66 | <0.001 |
| CK (U/L) | 130.20 (103.40, 196.50) | 128.60 (102.40, 184.40) | 758.95 (200.28, 1758.33) | <0.001 |
| CK-MB (U/L) | 12.60 (11.30, 15.20) | 12.50 (11.30, 14.90) | 24.90 (13.67, 41.85) | <0.001 |
| LDH (U/L) | 218.82 ± 79.01 | 215.29 ± 75.35 | 296.32 ± 111.13 | <0.001 |
| HBDH (U/L) | 177.24 ± 49.21 | 175.78 ± 48.27 | 209.33 ± 57.91 | <0.001 |
| Na (mmol/L) | 138.57 ± 1.76 | 138.58 ± 1.78 | 138.39 ± 1.14 | <0.001 |
| K (mmol/L) | 3.79 ± 0.17 | 3.79 ± 0.17 | 3.78 ± 0.15 | 0.039 |
| Cl (mmol/L) | 103.37 ± 1.97 | 103.40 ± 1.99 | 102.59 ± 1.35 | <0.001 |
| Ca (mmol/L) | 2.20 ± 0.07 | 2.20 ± 0.07 | 2.21 ± 0.07 | <0.001 |
| P (mmol/L) | 0.99 ± 0.09 | 0.99 ± 0.09 | 1.01 ± 0.11 | <0.001 |
| Lactate (mmol/L) | 2.52 ± 0.41 | 2.51 ± 0.41 | 2.83 ± 0.37 | <0.001 |
| Anion gap (mmol/L) | 12.38 ± 1.37 | 12.35 ± 1.36 | 13.05 ± 1.44 | <0.001 |
| Total carbon dioxide (mmol/L) | 22.78 ± 1.22 | 22.79 ± 1.22 | 22.73 ± 1.02 | 0.140 |
| NIHSS | 8.01 ± 2.94 | 7.85 ± 2.86 | 11.50 ± 2.51 | <0.001 |
| Gender, n(%) |  |  |  | <0.001 |
| Male | 10616 (49.47) | 10252 (49.95) | 364 (38.89) |  |
| Female | 10843 (50.53) | 10271 (50.05) | 572 (61.11) |  |
| Uremia (%) |  |  |  | <0.001 |
| No | 21269 (99.11) | 20352 (99.17) | 917 (97.97) |  |
| Yes | 190 (0.89) | 171 (0.83) | 19 (2.03) |  |
| DVT (%) |  |  |  | <0.001 |
| No | 20135 (93.83) | 19301 (94.05) | 834 (89.10) |  |
| Yes | 1324 (6.17) | 1222 (5.95) | 102 (10.90) |  |
| Fatty Liver (%) |  |  |  | <0.001 |
| No | 17214 (80.22) | 16419 (80.00) | 795 (84.94) |  |
| Yes | 4245 (19.78) | 4104 (20.00) | 141 (15.06) |  |
| Diabetes (%) |  |  |  | 0.497 |
| No | 14137 (65.88) | 13530 (65.93) | 607 (64.85) |  |
| Yes | 7322 (34.12) | 6993 (34.07) | 329 (35.15) |  |
| Hypertension (%) |  |  |  | 0.445 |
| No | 6708 (31.26) | 6426 (31.31) | 282 (30.13) |  |
| Yes | 14751 (68.74) | 14097 (68.69) | 654 (69.87) |  |
| Coronary Artery Disease (%) |  |  |  | <0.001 |
| No | 11787 (54.93) | 11195 (54.55) | 592 (63.25) |  |
| Yes | 9672 (45.07) | 9328 (45.45) | 344 (36.75) |  |
| Atrial Fibrillation (%) |  |  |  | 0.007 |
| No | 19416 (90.48) | 18593 (90.60) | 823 (87.93) |  |
| Yes | 2043 (9.52) | 1930 (9.40) | 113 (12.07) |  |
| Cerebral Herniation (%) |  |  |  | <0.001 |
| No | 21278 (99.16) | 20362 (99.22) | 916 (97.86) |  |
| Yes | 181 (0.84) | 161 (0.78) | 20 (2.14) |  |
| Hydrocephalus (%) |  |  |  | <0.001 |
| No | 21178 (98.69) | 20300 (98.91) | 878 (93.80) |  |
| Yes | 281 (1.31) | 223 (1.09) | 58 (6.20) |  |
| Hyperuricemia (%) |  |  |  | <0.001 |
| No | 19112 (89.06) | 18327 (89.30) | 785 (83.87) |  |
| Yes | 2347 (10.94) | 2196 (10.70) | 151 (16.13) |  |
| Hyperlipidemia (%) |  |  |  | <0.001 |
| No | 16990 (79.17) | 16204 (78.96) | 786 (83.97) |  |
| Yes | 4469 (20.83) | 4319 (21.04) | 150 (16.03) |  |
| Hypoproteinemia (%) |  |  |  | <0.001 |
| No | 19038 (88.72) | 18278 (89.06) | 760 (81.20) |  |
| Yes | 2421 (11.28) | 2245 (10.94) | 176 (18.80) |  |
| Frontal Lobe (%) |  |  |  | <0.001 |
| No | 20540 (95.72) | 19687 (95.93) | 853 (91.13) |  |
| Yes | 919 (4.28) | 836 (4.07) | 83 (8.87) |  |
| Parietal Lobe (%) |  |  |  | <0.001 |
| No | 20796 (96.91) | 19928 (97.10) | 868 (92.74) |  |
| Yes | 663 (3.09) | 595 (2.90) | 68 (7.26) |  |
| Temporal Lobe (%) |  |  |  | <0.001 |
| No | 20823 (97.04) | 19952 (97.22) | 871 (93.06) |  |
| Yes | 636 (2.96) | 571 (2.78) | 65 (6.94) |  |
| Occipital Lobe (%) |  |  |  | <0.001 |
| No | 21064 (98.16) | 20162 (98.24) | 902 (96.37) |  |
| Yes | 395 (1.84) | 361 (1.76) | 34 (3.63) |  |
| Insular Lobe (%) |  |  |  | 0404 |
| No | 21180 (98.70) | 20259 (98.71) | 921 (98.40) |  |
| Yes | 279 (1.30) | 264 (1.29) | 15 (1.60) |  |
| Basal Ganglia (%) |  |  |  | 0.011 |
| No | 20491 (95.49) | 19613 (95.57) | 878 (93.80) |  |
| Yes | 968 (4.51) | 910 (4.43) | 58 (6.20) |  |
| Capsula Interna (%) |  |  |  | 1.000 |
| No | 21445 (99.93) | 20509 (99.93) | 936 (100.00) |  |
| Yes | 14 (0.07) | 14 (0.07) | 0 (0.00) |  |
| Brainstem (%) |  |  |  | 0.217 |
| No | 21187 (98.73) | 20267 (98.75) | 920 (98.29) |  |
| Yes | 272 (1.27) | 256 (1.25) | 16 (1.71) |  |
| Epencephalon (%) |  |  |  | 0.763 |
| No | 21017 (97.94) | 20099 (97.93) | 918 (98.08) |  |
| Yes | 442 (2.06) | 424 (2.07) | 18 (1.92) |  |
| Paraventricular (%) |  |  |  | 0.196 |
| No | 20412 (95.12) | 19530 (95.16) | 882 (94.23) |  |
| Yes | 1047 (4.88) | 993 (4.84) | 54 (5.77) |  |
| Centrum Semiovale (%) |  |  |  | 0.278 |
| No | 20849 (97.16) | 19945 (97.18) | 904 (96.58) |  |
| Yes | 610 (2.84) | 578 (2.82) | 32 (3.42) |  |
| Thalamus (%) |  |  |  | 0.033 |
| No | 21222 (98.90) | 20303 (98.93) | 919 (98.18) |  |
| Yes | 237 (1.10) | 220 (1.07) | 17 (1.82) |  |
| Anterior Cerebral Artery (%) |  |  |  | 0.755 |
| 0 | 21185 (98.72) | 20262 (98.73) | 923 (98.61) |  |
| 1 | 274 (1.28) | 261 (1.27) | 13 (1.39) |  |
| Middle Cerebral Artery (%) |  |  |  | 0.397 |
| No | 20631 (96.14) | 19736 (96.17) | 895 (95.62) |  |
| Yes | 828 (3.86) | 787 (3.83) | 41 (4.38) |  |
| Posterior Cerebral Artery (%) |  |  |  | 0.899 |
| No | 21397 (99.71) | 20463 (99.71) | 934 (99.79) |  |
| Yes | 62 (0.29) | 60 (0.29) | 2 (0.21) |  |
| Vertebral Artery (%) |  |  |  | 0.779 |
| No | 20807 (96.96) | 19898 (96.95) | 909 (97.12) |  |
| Yes | 652 (3.04) | 625 (3.05) | 27 (2.88) |  |
| Basilar Artery (%) |  |  |  | 0.812 |
| No | 21268 (99.11) | 20341 (99.11) | 927 (99.04) |  |
| Yes | 191 (0.89) | 182 (0.89) | 9 (0.96) |  |
| Common Carotid Artery Plaque (%) |  |  |  | 0.406 |
| No | 16636 (77.52) | 15900 (77.47) | 736 (78.63) |  |
| Yes | 4823 (22.48) | 4623 (22.53) | 200 (21.37) |  |
| Internal Carotid Artery Plaque (%) |  |  |  | 0.105 |
| No | 19997 (93.19) | 19137 (93.25) | 860 (91.88) |  |
| Yes | 1462 (6.81) | 1386 (6.75) | 76 (8.12) |  |
| External Carotid Artery Plaque (%) |  |  |  | 0.439 |
| No | 21258 (99.06) | 20333 (99.07) | 925 (98.82) |  |
| Yes | 201 (0.94) | 190 (0.93) | 11 (1.18) |  |
| Subcortical Lobe (%) |  |  |  | 0.003 |
| No | 19016 (88.62) | 18215 (88.75) | 801 (85.58) |  |
| Yes | 2443 (11.38) | 2308 (11.25) | 135 (14.42) |  |
| Anterior Circulation (%) |  |  |  | 0.391 |
| No | 20046 (93.42) | 19178 (93.45) | 868 (92.74) |  |
| Yes | 1413 (6.58) | 1345 (6.55) | 68 (7.26) |  |
| Posterior Circulation (%) |  |  |  | 0.067 |
| No | 16958 (79.03) | 16196 (78.92) | 762 (81.41) |  |
| Yes | 4501 (20.97) | 4327 (21.08) | 174 (18.59) |  |

Note: Continuous variables were described by mean ± standard deviation or median (interquartile range) and categorical variables were presented by number (percentage).

Abbreviations: PSE, Post-stroke epilepsy; WBC, White Blood Cell; RBC, Red Blood Cell; HbA1c, Glycated Hemoglobin; CRP, C-Reactive Protein; TG, Triglyceride; LDL-c, Low-Density Lipoprotein cholesterol; HDL-c, High-Density Lipoprotein cholesterol; AST, Aspartate Aminotransferase; ALT, Alanine Aminotransferase; PT, Prothrombin Time; APTT, Activated Partial Thromboplastin Time; TT, Thrombin Time; INR, International Normalized Ratio; CK, Creatine Kinase; CK-MB, Creatine Kinase-MB Isoenzyme; LDH, Lactate Dehydrogenase; HBDH, Hydroxybutyrate Dehydrogenase; Na, Sodium; K, Potassium; Cl, Chloride; Ca, Calcium; P, Phosphorus; NIHSS, National Institutes of Health Stroke Scale; DVT, deep vein thrombosis.
